# Socioeconomic inequalities in mental health and wellbeing among UK students during the COVID-19 pandemic: Clarifying underlying mechanisms

**DOI:** 10.1371/journal.pone.0292842

**Published:** 2023-11-01

**Authors:** Isla Dougall, Milica Vasiljevic, Maja Kutlaca, Mario Weick

**Affiliations:** Department of Psychology, Durham University, Durham, United Kingdom; St John’s University, UNITED STATES

## Abstract

Universities are seeing growing numbers of students with poor mental health and wellbeing. Given that lower socioeconomic status (SES) students typically have poorer mental health and wellbeing than their peers, this may be, in part, caused by an increase in the number of students attending university from lower SES backgrounds. However, less is known about how socioeconomic inequalities in mental health and wellbeing persist within university communities. Research investigating psychosocial factors that contribute to socioeconomic disparities in mental health and wellbeing suggests perceived control, inclusion, and perceived worth to be important underlying mechanisms. However, another strand of research suggests perceived competence may also play a mediating role in this relationship. Consequently, the present research seeks to examine fulfilment of perceived control, inclusion, perceived worth, and competence needs as potential mediators in the relationship between subjective SES and mental health and wellbeing in university students. Below, we report the results of a cross-sectional survey conducted among university students (*n* = 811) in the UK during a period of COVID-19 restrictions. In line with prior research, we found evidence of socioeconomic inequalities in mental health and wellbeing among students. Further, we found subjective SES predicted perceptions of control, inclusion, and competence. In turn, perceived control and competence predicted both positive and negative mental health and wellbeing, whilst inclusion predicted positive mental health and wellbeing only. Unexpectedly, we found no evidence that perceived worth acts as a mediator in this relationship, independently of perceived control, inclusion, and competence. As academic institutions continue to pursue policies to ‘widen participation’, they also have a responsibility to understand how socioeconomic inequalities in mental health and wellbeing are perpetuated within the university community. Research in this area marks a first step to improve socioeconomic equality within Higher Education.

## Introduction

Around the world, universities are growing increasingly concerned due to high proportions of students with poor mental health and wellbeing [1–3]. In the UK, more than two-fifths (42.3%) of students in Higher Education (HE) have experienced a serious psychological issue for which they needed professional help [4]. In Australia and the US, prevalence of psychological distress among HE students is similarly high (47.6% and 40.8%, respectively) [5, 6]. Notably, it is thought that schemes that aim to ‘widen participation’ to groups that are traditionally underrepresented in HE, such as those from lower socioeconomic backgrounds, may have contributed to the growing number of students with poor mental health and wellbeing among students [7]. Research supports this, and finds socioeconomic inequalities in mental health and wellbeing within universities, whereby those with lower socioeconomic status (SES) have poorer mental health and wellbeing [8]. However, the underlying mechanisms that contribute towards this pattern remain unclear. For example, whilst research has suggested that psychosocial factors such as perceived control, inclusion, and perceived worth are consequential to mental health and wellbeing, the role of perceived competence remains uncertain. The present research will examine socioeconomic inequalities in mental health and wellbeing among university students and aims to clarify the underlying psychosocial mechanisms that act in this relationship.

Existing research suggests that lower (*vs*. higher) SES students experience poorer mental health and wellbeing on measures such as anxiety and depression [9–11]. Within the general population, whilst some research has suggested little or no relationship between socioeconomic status and mental health and wellbeing [e.g., 12, 13], substantially more research has suggested that higher socioeconomic status is related to better mental health and wellbeing [e.g., 14]. In trying to explain this, research has adopted a need-fulfilment perspective, where the fulfilment of a select few psychosocial needs are considered crucial to mental health and wellbeing [e.g., 15, 16]. *Perceived control*, also referred to as autonomy, is a construct that reflects whether life outcomes are perceived to be determined by an individual, or by something external to an individual such as luck or fate [17]. Research suggests that low perceptions of control in life can have a detrimental impact on mental health and wellbeing, and can explain, at least in part, socioeconomic variation in mental health and wellbeing [18]. *Inclusion* is rooted in social liking and acceptance [19] and research suggests that social support can buffer the detrimental effects of lower SES on mental ill-health [20]. Finally, *perceived worth*, also referred to as status, is rooted in respect and admiration [19] and research suggests that feeling respected can mediate the impact of SES on subjective wellbeing and quality of life [21, 22].

A large-scale survey of participants from the general population of 123 countries around the world examined the association between need-fulfilment and mental health and wellbeing, and explored autonomy (*i*.*e*., perceived control), social (*i*.*e*., inclusion), respect (*i*.*e*., perceived worth) and mastery needs (*i*.*e*., competence), among others. In this research, autonomy (*i*.*e*., perceived control), social (*i*.*e*., inclusion), and respect (*i*.*e*., perceived worth) needs emerged as the only needs, alongside basic needs for food and shelter, that accounted for differences in mental health and wellbeing [23]. Research examining these factors in parallel in HE settings suggests that perceived control, inclusion, and perceived worth uniquely and jointly contribute to socioeconomic inequalities in mental health and wellbeing. Put simply, relative to their higher SES peers, students with lower SES report feeling less in control, less liked, and less respected, and this in turn may be detrimental for their mental health and wellbeing [24].

Other lines of research draw upon Basic Psychological Needs Theory to explain socioeconomic inequalities in mental health and wellbeing. Basic Psychological Needs Theory is one of six theories nested within Self-Determination Theory. This theory suggests there are a set of three psychosocial needs that are essential for an individuals’ mental health and wellbeing [16]. Basic Psychological Needs Theory suggests that autonomy (*i*.*e*., perceived control), relatedness (*i*.*e*., inclusion), and competence drive socioeconomic disparities in mental health and wellbeing [25, 26]. C*ompetence* here refers to a sense of accomplishment and growing mastery in one’s activities [25]. Whilst research rooted in Basic Psychological Needs Theory assumes that *competence* is essential to mental health and wellbeing, it does not recognise *perceived worth* as a psychological need that operates independently from other essential needs (*i*.*e*., perceived control, inclusion, and competence). In contrast, Tay and Diener [23] found ‘respect’ (*i*.*e*., perceived worth) to be more consequential to wellbeing than ‘mastery’ [i.e., competence; 23] in the general population. This is consistent with theoretical work that has made a strong case that perceived worth is a fundamental human motive that impacts mental health and wellbeing [27]. Whilst competence centres around self-perceptions of capability and accomplishment, perceived worth considers respect and admiration afforded by others. Therefore, the question remains; which psychosocial needs contribute to socioeconomic inequalities in mental health and wellbeing among university students? In particular, what are the roles of competence and perceived worth?

This research question became particularly poignant during the COVID-19 pandemic. During this time, universities pivoted to remote teaching and many university students returned to their family homes to learn online. These changes may have particularly impacted competence-based needs as students were challenged to find ‘a new way of working’. This might have been more difficult for students from lower SES backgrounds as they were more likely to face additional challenges that made it more difficult to maintain their studies including, but not limited to, finding an appropriate space to work and sourcing necessary technology [28]. However, perceived worth may also have been important during this time. For example, research suggests that low-status groups, such as lower SES students, want to feel respected and empowered, more so than high-status groups [29]. During the COVID-19 pandemic, changes to the ways that students socialised and interacted may have influenced the extent to which perceived worth could be inferred from others. This may have been particularly consequential for lower SES students. Considering this, it is important to review the roles of perceived control, inclusion, perceived worth, and competence in explaining the socioeconomic patterning of mental health and wellbeing among students during this time.

Whilst one line of research suggests that perceived control, inclusion, and perceived worth are important underlying mechanisms in the relationship between subjective SES and mental health in students, another line of research suggests that competence is also an essential underlying mechanism. Consequently, the present research seeks to examine fulfilment of perceived control-, inclusion-, perceived worth-, and competence-based needs as potential mediators in the relationship between subjective SES and mental health and wellbeing among university students. Below, we report the results of a cross-sectional survey conducted among university students in the UK during a period of COVID-19 restrictions, testing the hypothesis that subjective SES predicts the fulfilment of perceived control-, inclusion-, perceived worth- and competence-based needs, which in turn predicts mental health and wellbeing.

## Method

### Participants

Data were collected between December 2020 and March 2021 during a time of COVID-19 restrictions in the UK [for details of restrictions, see 30]. University students were recruited online using an undergraduate participant pool to capture Psychology students completing studies for course credit. Additionally, university students from around the UK were recruited via social media posts. As compensation, students were offered entry into a prize draw to win shopping vouchers (up to £50). Finally, students were recruited from Prolific and were paid £1.25 for taking part. Overall, 1,165 participants started the survey, and of those, 908 (77.9%) reached the end. Of the participants who reached the end of the survey, 90 (9.9%) were excluded due to incomplete data (see Missing Data section in Results) and 7 participants (0.8%) were excluded for failing a planned attention check. Our final sample consisted of 811 participants from 90 universities around the UK. The majority of participants were recruited from Durham University, UK (*n* = 615, 75.9%). Participant demographic characteristics are reported in Table 1 and demographics of the excluded sample are reported in S1 Appendix. The mean age of participants was 21.23 years (*SD* = 5.42).

**Table 1:** Demographic characteristics of retained participant sample (*n* = 811).

Characteristic	*n*	%
Gender		
Female	588	72.5
Male	223	27.5
Ethnicity		
White	593	73.1
Asian/Asian British	142	17.5
All Other Ethnic Groups	76	9.4
Parent/Guardian Household Income		
<£15,500	93	11.5
£15,500 - £24,999	90	11.1
£25,000 - £40,000	149	18.4
>£40,000	479	59.1
Recruitment Method		
Psychology Participant Pool	429	52.9
Social Media	222	27.4
Prolific	160	19.7

We aimed to recruit around 1,000 participants, or as many participants as possible before the end of the academic term in March 2021, given that SEM requires large sample sizes. Power analysis is discussed in further detail in the Analytical Strategy section below.

### Procedure

Ethical approval for the present study was granted by Durham University Department of Psychology Ethics Sub-committee. The present research was preregistered using AsPredicted (https://aspredicted.org/WC9_5T9) and the questionnaire was administered online using Qualtrics (www.qualtrics.com).

### Materials

#### Demographic characteristics

Participants reported their gender, age, ethnicity, parent/guardian household income, parent/guardian educational level, occupation of chief income earner in parent/guardian household, the number of people in their household, and whether they had undertaken a period of self-isolation since the beginning of the academic year (yes/no). These demographic items served as control variables in sensitivity analyses (see S5 Appendix).

#### Subjective Socioeconomic Status (SES)

The McArthur ladder was used to assess subjective SES, with higher rungs indicating higher SES [31]. Because participants were students, we asked them to indicate where they felt they stood on the ladder in terms of their *family’s* money, education, and occupational prestige (0 to 10).

Participants also reported economic, social and cultural capital via three questions based on Bourdieu’s theoretical framework [32]. Economic capital was defined as income, savings, the value of your family’s home and your family’s wealth. Social capital was defined as the number of people you know and the status of those people. Cultural capital was defined as the extent and nature of your cultural interests, activities, and hobbies (0 = *lowest capital* to 100 = *highest capital*).

#### Perceived control

The perceived control scale was comprised of two statements adapted from Cichocka et al. [33]; “I have great control over my life” and “I have great influence on my fate.” In addition, we added four items; “I am able to decide what happens to me,” “I am able to control the important things in my life,” “I am able to control how I spend my time,” and “I am free to do what I want” (1 = *strongly disagree*; 7 = *strongly agree*).

#### Inclusion

We adapted the 9-item scale developed by Mahadevan et al. [19]. The scale began with the stem “Most of the time I feel that other students…” and participants were asked to assess the following statements: “like me as a person,” “feel warmly towards me,” “consider me to be a nice person to have around,” and “don’t like me (R),” “include me in their social activities,” “are happy for me to belong to their social groups,” “accept me,” “see me as fitting in,” and “would be willing to be friends with me” (1 = *strongly disagree*; 7 = *strongly agree*).

#### Perceived worth

We adapted the 8-item scale developed by Mahadevan et al. [19] which began with the stem “Most of the time I feel that other students…” Participants were asked to assess the following statements: “respect my achievements,” “value my opinions and ideas,” “think highly of my abilities and talents,” “admire me,” “consider me a success,” “look up to me,” “see me as an important person,” and “consider me a high-status individual” (1 = *strongly disagree*; 7 = *strongly agree*). As reported in the S2 Appendix, one item (“value my opinions and ideas”) was removed following EFA as it had a high cross loading. The remaining items loaded onto one factor.

#### Competence

We assessed competence using six items adapted from the Basic Psychological Need Satisfaction Scale [34, 35]. Items included “I often have not felt very capable (R),” “I have felt a sense of accomplishment from what I do,” “I have not felt very competent (R),” “I have not had much of a chance to show how capable I am (R),” “People I know have told me I am good at what I do,” and “I have been able to learn interesting new skills” (1 = *strongly disagree*; 7 = *strongly agree*). The latter three items were removed following EFA due to cross loading with other factors. See S2 Appendix for complete analysis.

#### Mental health and wellbeing

We developed a mental health and wellbeing scale as existing scales did not (1) include items specific to student mental health and wellbeing (e.g., anxiety and stress) [36, 37], and (2) capture hedonic and eudaimonic attributes of mental health and wellbeing [38]. This scale used 12 items to measure various attributes of mental wellbeing and physical health. Four items measured subjective wellbeing and were adapted from the measure used by the Office for National Statistics [39]; “Since the start of the academic year in October, how often have you felt satisfied with your life?”, “Since the start of the academic year in October, how often have you felt like the things you do in your life are worthwhile?”, “Overall, how happy did you feel yesterday?”, and “Overall, how anxious did you feel yesterday?”. We adapted two items from the Questionnaire for Eudaimonic Well-being [40]; “Since the start of the academic year in October, how often have you felt like you had your purpose in life?” and “Since the start of the academic year in October, how often have you felt fulfilled by the activities that you engaged in?”. We adapted two items from the Perceived Stress Scale (PSS) [41]; “Since the start of the academic year in October, how often have you felt nervous and stressed?” and “Since the start of the academic year in October, how often have you felt that you were effectively coping with important changes that were occurring in your life?”.

We also included two items that we designed; “Since the start of the academic year in October, how often have you felt emotionally exhausted?” and “Since the start of the academic year in October, how often did you worry?”. Previous research has also suggested a single-item to assess problematic worry [42]. Lastly, we adapted two items from the PROMIS global physical and mental health scales [43]; “How would you rate your mental health, including your mood and ability to think, since the lockdown began in March 2020?” and “How would you rate your physical health, since the lockdown began in March 2020?”. All mental health and wellbeing items used response scales that ranged from 0 (*not at all/poor*, *as appropriate*) to 10 (*completely/excellent*, *as appropriate*). EFA highlighted high cross loadings for the two items from the PROMIS global physical and mental health scales, so these items were removed. The remaining 10 items loaded across two factors; positive wellbeing–which included 6 items relating to happiness, coping, purpose, fulfilment, worthwhileness, and life satisfaction, and negative wellbeing–which included 4 items relating to anxiety, worry, stress, and emotional exhaustion. Full details are provided in S2 Appendix.

#### Additional information

Unless stated otherwise in the sections above, items from each scale loaded onto one factor. Complete EFA analyses for each scale are reported in S2 Appendix. Within each scale, the presentation of the items was randomised across participants. Further, the scales were randomly presented according to the following rules: the mental health and wellbeing scale was presented first or last. The subjective SES scale and the perceived control, inclusion, perceived worth, and competence scales were presented before or after the mental health and wellbeing scale; finally, an attention check was shown between the perceived control, inclusion, perceived worth, and competence scales. The attention check was worded as follows, “This is an attention check. Please select ‘never’” (*1 = never to 5 = always*).

Additional data were collected that were intended for use in other research projects; whether participants knew anyone that had had COVID-19, the duration of their most recent isolation period, and perceptions of perceived control, inclusion, and perceived worth afforded by policymakers. In the present manuscript, we report all data from items pertaining to subjective SES, perceived control, competence, wellbeing, and perceived worth and inclusion afforded by other students.

### Analytical strategy

Data were analysed using R [v4.1.1; 44]. In our preregistration we specified that we would use the PROCESS macro to conduct ordinary least squares (OLS) path analysis [45] (see S3 Appendix for futher details). However, the results of the mental health and wellbeing EFA suggested a two-factor solution. As PROCESS does not allow for this type of analysis, we decided to use Structural Equation Modelling (SEM). As there are differences in the sample size requirements for PROCESS and SEM, we conducted post-hoc power analyses which suggested that we were adequately powered to reject a misspecified model based on the RMSEA index of model fit (effect size as the threshold value required to detect ‘good’ model fit, RMSEA = 0.06, *α* = 0.05, *n* = 811 and df = 644, 1-β = >.99) [46].

A series of preliminary checks were conducted on the data to determine whether the basic assumptions recommended for structural equation modelling (SEM) were met. We examined the correlations between items on the same latent factor, looking for correlations < 0.5. We assessed extreme collinearity between the items to confirm that none correlated > 0.85 and established that the relationships between the X to M and M to Y variables were linear. Finally, we tested for normality of distribution. The skew and kurtosis was below commonly accepted thresholds for all items (< 3 and < 10, respectively) [47]. However, a Mardia test of multivariate normality of distribution indicated non-normal distribution. For this reason, we employed maximum likelihood (ML) estimation with bootstrapping (5,000 samples) [48].

First, we conducted Pearson’s correlations on the data to identify overarching relationships between our variables. We conducted confirmatory factor analysis to determine goodness-of-fit of the model suggested by exploratory factor analysis. We then analysed the primary–hypothesised–model, which included subjective SES, perceived control, inclusion, perceived worth, competence, and mental health and wellbeing. Second, to provide an indication of sensitivity, we analysed the primary model alongside additional control variables; gender, age, ethnicity, the number of people in their household, and whether they had self-isolated since the beginning of the academic year (yes/no). Finally, we provide a comparison model that uses *objective* measures of SES. For conciseness, the additional models are reported in brief below, with further detail provided in S5 and S6 Appendices.

Participants with missing data were removed listwise for two reasons; (1) our method of estimation required complete cases (maximum likelihood estimation with bootstrapping), and (2) the proportion of incomplete data for the items included in our model (0.2%) was far below the suggested threshold [5%; 47]. In our analysis, the variance of one indicator in each latent factor was fixed to 1 and all latent factors were allowed to correlate freely with each other.

To evaluate model fit, we report relative indices of fit (CFI and TLI), alongside an absolute index of fit (RMSEA) [49]. Research has suggested the following “rules of thumb” to evaluate model fit; CFI and TLI close to or above0.95, and RMSEA close to or below 0.06 [49]. A non-significant chi-square test also suggests good model fit, although the usefulness of this measure is limited in models with large sample sizes [47]. Similarly, we also note that many researchers have cautioned against a strict reliance of measures of model fit to indicate an acceptable model [47, 50].

## Results

Descriptive statistics for, and correlations between, the mean-scores of each scale are presented in Table 2. (*NB*. The statistics presented in this table illustrate the overall pattern of relationships between the scales, however the SEM reported in the following analysis used individual scale items). Lower (*vs*. higher) subjective SES students experienced greater levels of anxiety, stress, and worry (negative mental health and wellbeing; *r* = -.16, *p* < .001), and lower fulfilment, purpose, and life satisfaction (positive mental health and wellbeing; *r* = .30, *p* < .001).

**Table 2:** Means, standard deviations, and correlations with confidence intervals.

Variable	*M*	*SD*	1	2	3	4	5	6
1. Subjective SES	5.66	1.69						
2. Perceived Control	3.89	1.34	.23**					
			[.16, .29]					
3. Inclusion	5.09	1	.33**	.23**				
			[.27, .39]	[.16, .29]				
4. Perceived Worth	3.87	1.08	.38**	.29**	.54**			
			[.32, .43]	[.22, .35]	[.49, .58]			
5. Competence	3.42	1.27	.15**	.45**	.22**	.36**		
			[.08, .22]	[.39, .50]	[.15, .29]	[.30, .42]		
6. Positive Wellbeing	5.06	1.78	.30**	.57**	.38**	.39**	.60**	
			[.23, .36]	[.53, .62]	[.31, .43]	[.33, .45]	[.55, .64]	
7. Negative Wellbeing	6.57	1.86	-.16**	-.44**	-.21**	-.24**	-.49**	-.51**
			[-.22, -.09]	[-.50, -.39]	[-.28, -.15]	[-.30, -.17]	[-.54, -.43]	[-.56, -.46]

Note. M and SD are used to represent mean and standard deviation, respectively. *Wellbeing* indicates mental health and wellbeing. Subjective SES and wellbeing had a response scale from 0 to 10. Perceived control, inclusion, and perceived worth scales had a response scale from 1 to 7. Values in square brackets indicate the 95% confidence interval.

** *p* < .001

* *p* < .05

### Measurement model

A series of preliminary checks were conducted on the data to determine whether the basic assumptions recommended for SEM were met (see S4 Appendix for complete details). A Mardia test of multivariate normality indicated non-normal distribution, so we employed maximum likelihood (ML) estimation with bootstrapping (5,000 samples) [48].

Our measurement model specified seven latent factors–subjective SES, perceived control, inclusion, perceived worth, competence, positive mental health and wellbeing, and negative mental health and wellbeing. Each of these latent factors had between four and nine items. We used confirmatory factor analysis to determine the fit of this model. The goodness-of-fit indices suggest that the fit of the measurement model was fair; the model met the threshold for RMSEA and was close to the threshold for CFI and TLI. One item–concerning cultural capital from the subjective SES factor–had a factor loading below the recommended threshold of 0.6 [51]. For this reason, it was removed, and the revised model had significantly improved fit. Results of the initial and revised model are provided in Table 3. Scale items, standardised factor loadings, composite reliability, average variance explained (AVE) and Cronbach’s alpha for the measurement model are presented in Table 4; the full wording of all items is presented in S2 Appendix.

**Table 3:** Measurement models goodness-of-fit indices.

Model	χ^2^	df	Δχ2	Δdf	RMSEA	CFI	TLI
Null Model	20185.16**	741	–	–	–	–	–
Initial Model	2042.53**	681	–	–	0.050	0.930	0.924
Revised Model	1852.73**	644	189.81**	37	0.048	0.937	0.931

** *p* < .001

**Table 4:** Standardised factor loadings, composite reliability, average variance explained, and Cronbach’s alpha for the measurement model.

Construct and indicators	Standardised Loadings	Composite Reliability	AVE	α
Subjective SES		0.817	0.603	0.80
SES1—Subjective SES Ladder	0.889			
SES2—Economic Capital	0.857			
SES3—Social Capital	0.609			
Perceived Control		0.888	0.571	0.89
PC1—Ctrl Life	0.850			
PC2—Influence	0.745			
PC3—Decide	0.787			
PC4—Ctrl Important Things	0.823			
PC5—Ctrl Time	0.633			
PC6—Free	0.699			
Inclusion		0.933	0.614	0.93
Incl1—Liking	0.819			
Incl2—Warmth	0.817			
Incl3—Friendship	0.846			
Incl4—Belonging	0.857			
Incl5—Fitting In	0.777			
Incl6—Including	0.770			
Incl7—Niceness	0.773			
Incl8—Disliking (R)	0.587			
Incl9—Acceptance	0.793			
Perceived Worth		0.907	0.584	0.91
PW1—Respect	0.683			
PW2—Talent	0.752			
PW3—Admiration	0.829			
PW4—Importance	0.750			
PW5—Success	0.761			
PW6—Look up to	0.844			
PW7—High Status	0.716			
Competence		0.803	0.580	0.79
Comp1—Competent	0.827			
Comp2—Accomplishment	0.609			
Comp3—Not Capable (R)	0.831			
Positive Wellbeing		0.890	0.578	0.89
WBP1—Life Satisfaction	0.845			
WBP2—Happiness	0.598			
WBP3—Purpose	0.811			
WBP4—Worthwhile	0.818			
WBP5—Fulfilment	0.722			
WBP6—Coping	0.740			
Negative Wellbeing		0.840	0.568	0.84
WBN1—Stress	0.895			
WBN2—Worry	0.817			
WBN3—Emotional Exhaustion	0.798			
WBN4—Anxiety	0.580			

*Note*. *AVE* indicates Average Variance Explained. *Wellbeing* indicates mental health and wellbeing.

### Structural model

**Fig 1:**
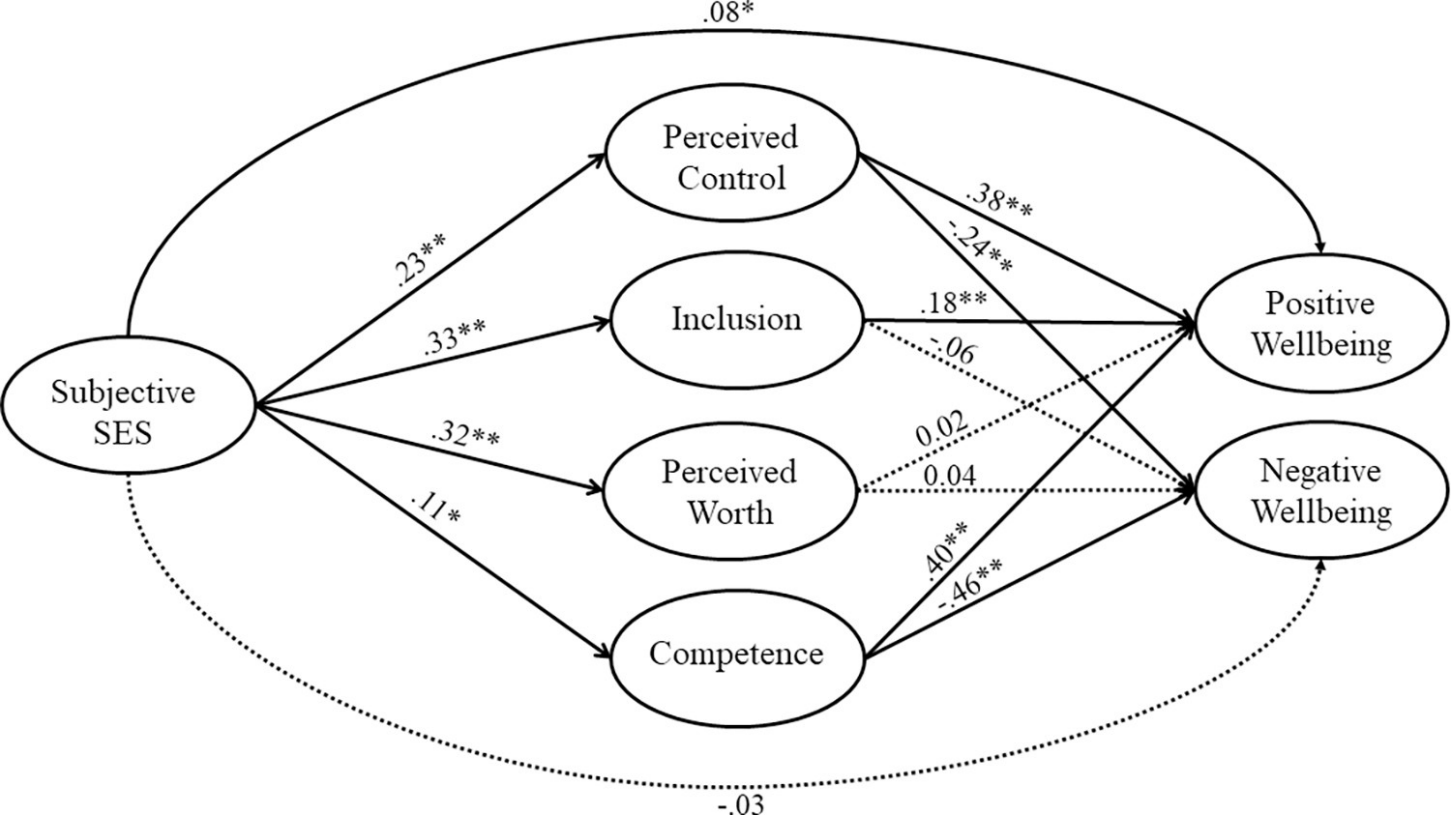
Standardised path estimates for primary model. *Note*. *Wellbeing* indicates mental health and wellbeing. Mediators were allowed to covary, as were the two wellbeing variables. Dashed lines are used to emphasise non-significant paths. **p < .001 * *p* < .05.

We tested the structural model illustrated in Fig 1. Model-fit was adequate; RMSEA passed the threshold indicating good fit, and values of CFI and TLI were close to the recommended threshold (χ2 (644, 811) = 1852.73, *p* < .001, RMSEA = 0.048, CFI = 0.937, TLI = 0.931).

### Indirect effects

We hypothesised there would be indirect effects of subjective SES on mental health and wellbeing through perceived control, inclusion, perceived worth, and competence. Supporting our hypothesis, we found significant indirect effects through perceived control and competence for both positive and negative mental health and wellbeing. Lower (*vs*. higher) subjective SES was linked to lower perceived control and lower competence, both of which were associated with lower levels of positive mental health and wellbeing, and higher levels of negative mental health and wellbeing.

Additionally, we found an indirect effect via inclusion, but only for positive mental health and wellbeing, indicating partial support for our hypothesis. Lower (*vs*. higher) subjective SES was associated with lower perceived inclusion, which was linked to lower levels of fulfilment, purpose, and life satisfaction. Finally, contrary to our hypothesis, we found no support for an indirect effect via perceived worth. Whilst there was a significant association between subjective SES and perceived worth in the SEM model, we found no evidence of a significant association between perceived worth and mental health and wellbeing, independent of other mediators. Indirect effects are presented in Table 5 and path estimates can be found in Fig 1.

**Table 5:** Indirect and total effects of hypothesised mediators in primary model.

Parameter	Unstandardised Coefficient (b)	SE	Standardised Coefficient (β)
Indirect Effects			
SES -> Perceived Control -> Positive Wellbeing	0.107	0.021	0.085**
SES -> Perceived Control -> Negative Wellbeing	-0.065	0.016	-0.055**
SES -> Inclusion -> Positive Wellbeing	0.074	0.017	0.059**
SES -> Inclusion -> Negative Wellbeing	-0.025	0.017	-0.021
SES -> Perceived Worth -> Positive Wellbeing	0.010	0.016	0.008
SES -> Perceived Worth -> Negative Wellbeing	0.014	0.018	0.012
SES -> Competence -> Positive Wellbeing	0.056	0.020	0.045*
SES -> Competence -> Negative Wellbeing	-0.060	0.023	-0.051*
Total Effects			
SES -> Positive Wellbeing	0.342	0.050	0.271**
SES -> Negative Wellbeing	-0.172	0.047	-0.145**

*Note*. *SES* indicates subjective SES. *Wellbeing* indicates mental health and wellbeing.

** p < .001

* *p* < .01

### Sensitivity analysis

To provide sensitivity analyses, we analysed the above primary model but included a series of demographic covariates; gender, age, ethnicity, the number of people currently living in their household, and whether participants had self-isolated since the beginning of the academic term. Model fit was fair (χ2 (836, 811) = 2279.97, *p* < .001, RMSEA = 0.046, CFI = 0.926, TLI = 0.918). As shown more fully in S5 Appendix, the overall pattern of results was the same as the primary model in terms of statistically significant paths and indirect effects.

### Further analysis

We conducted further analysis that employed objective measures of SES, rather than subjective measures. We used measures of (1) parent/guardian household income, (2) parent/guardian educational level, and (3) occupation of chief income earner in parent/guardian household. These three items acted as indicator variables for the objective SES latent factor. Objective SES correlated substantively with subjective SES (*r* = 0.63, *p* < .001; calculated using mean scores).

Fit was fair for this model (χ2 (644, 701) = 1620.26, *p* < .001, RMSEA = 0.047, CFI = 0.939, TLI = 0.934). The pattern of result remained the same as the model with subjective SES regarding indirect effects via perceived control, inclusion, and perceived worth. However, in the objective SES model, we found no significant indirect effect via competence. Whilst there was a significant association between competence and both positive and negative mental health and wellbeing, there was no significant association between objective SES and competence. These results are presented in full in S6 Appendix.

## Discussion

In the present research, we examined factors that contribute to socioeconomic inequalities in mental health and wellbeing among UK university students during COVID-19 restrictions. In particular, we sought to clarify the role of competence as a mediator, alongside perceived control, inclusion, and perceived worth, in the relationship between subjective SES and mental health and wellbeing. In a large cross-sectional sample of university students, we found evidence of socioeconomic inequalities in mental health and wellbeing, whereby subjective SES was associated with positive (*r* = .30, *p* < .001) and negative (*r* = -.16, *p* < .001) mental health and wellbeing. Socioeconomic inequalities in mental health and wellbeing are well established among the general population [14], and previous research suggests socioeconomic disparities are also found within university student populations [8, 24]. The present research replicates these findings and extends previous work as it distinguishes between positive (*e*.*g*., life satisfaction, fulfilment, happiness) and negative (*e*.*g*., stress, worry, anxiety) aspects of mental health and wellbeing, and suggests socioeconomic inequalities in both aspects.

Further, in the present research, we found indirect effects via perceived control, inclusion, and competence. Put simply, subjective SES predicted feelings of control, inclusion, and competence. In turn, perceived control and competence predicted both positive and negative mental health and wellbeing, whilst inclusion predicted positive mental health and wellbeing only. This aligns with previous work that has drawn on Basic Psychological Needs Theory showing that the fulfilment of autonomy (*i*.*e*., perceived control), relatedness (*i*.*e*., inclusion), and competence needs can partially explain socioeconomic differences in mental health and wellbeing among students [25, 26].

Unexpectedly, we found no evidence that perceived worth acts as a mediator in this relationship, independently of perceived control, inclusion, and competence. Whilst the relationship between SES and perceived worth was significant, the association between perceived worth and mental health and wellbeing was non-significant in our model. This extends and qualifies previous research that has suggested the fulfilment of perceived worth as an explanation for socioeconomic disparities in mental health and wellbeing among university students [21, 24]. Given that the addition of competence is the key feature differentiating the present research from previous work, we suggest that, in the present sample, competence accounts for some of the association between perceived worth and mental health and wellbeing.

Imposterism could be one factor that explains why competence appears to be particularly consequential in the present sample of university students. Imposterism describes unfounded feelings of inadequacy, which are socially patterned and particularly prevalent among students from a lower (*vs*. higher) SES background [52]. Another explanation relates to the fact that following the introduction of substantive student fees, many students feel under pressure to perform well. Lower (*vs*. higher) SES students not only tend to accrue more debt [53], but as they are often part of the first generation to attend university, they may feel under particular pressure to perform well. Yet, lower (*vs*. higher) SES students also tend to have poorer academic performance at university [54]. The combination of career ambitions, academic performance, and debt could be another reason why competence is fundamental to students’ perceived worth and may account for the link between perceived worth and mental health and wellbeing.

### Implications for policy and practice

Building on previous work, the present research suggests that there may be opportunities to reduce socioeconomic inequalities in mental health and wellbeing among university students via strategies that improve perceptions of control, inclusion, and competence. For example, strategies to encourage learners’ perceived control include allowing students to shape the curriculum and assessments [55–57], and even allowing students to determine the consequences for poor conduct [58]. Such initiatives can benefit students’ approaches to learning, and crucially, perceptions of their own capabilities [e.g., 56]. The Stepchange: Mentally Healthy Universities report makes similar recommendations, arguing that students should be involved in the development of mental health policies and support services at their universities, empowering students to take responsibility for their own mental health and wellbeing [59]. These steps would allow students to develop a sense of control over their future and their mental health and wellbeing.

Additionally, we could consider perceived control in relation to students’ general livelihood. In recent years, the maintenance loan available to students has been deemed insufficient, particularly for students from lower SES backgrounds [e.g., 60]. If more funding was made available, this would likely benefit the latter students’ mental health and wellbeing by giving students greater freedom in deciding where they would like to live, whether to work part-time, and whether to engage in their university’s extracurricular activities, for example. Currently, many of these decisions are not available to lower SES students due to financial restrictions and the hidden costs of attending university [61]. Increased perceptions of control over life decisions such as these would likely benefit the mental health and wellbeing of lower SES students.

To consider inclusion, previous research suggests the use of peer-mentoring schemes to improve outcomes for lower SES university students [62, 63]. If these schemes increased a sense of community and perceived inclusion among peers, they may benefit students’ mental health and wellbeing. Further, lower SES students are often both time- and money-poor compared to their peers. Research suggests that strategies to bridge these gaps such as subsidising social events and providing accessible on-campus childcare and family accommodation, may improve perceived inclusion among these students as they are able to engage more fully in university life [64]. This illustrates the variety of ways that perceived exclusion can manifest among lower SES students, but also illustrates the variety of strategies that could be used to improve mental health and wellbeing in this group.

Competence derives from a combination of perceived ability and demands in any particular situation. Perceptions of competence may be improved by ensuring that students feel adequately supported throughout their studies, which, as the present work suggests, may in turn reduce inequalities in mental health and wellbeing. Importantly, universities would do well to challenge the deficit-discourse that often surrounds lower SES students, in which they are associated with academic struggle and failure [e.g., 65, 66]. Instead, universities can acknowledge background-specific strengths of lower SES students and the benefits that diverse perspectives bring to universities [67–69]. Reflecting on, and highlighting, the strengths and capabilities of lower SES students may boost perceived competence, which in turn would improve their mental health and wellbeing.

### Strengths and limitations

Key strengths of the present work include the large sample size of UK students (*n* = 811) which allowed us to employ SEM. Further, our chosen measures were fairly comprehensive. For example, our measure of subjective SES included aspects of economic and social capital, in addition to the commonly used ladder measure. Further, our measures of mental health and wellbeing included attributes of both wellness and illness that would be relevant to a student population.

The primary limitation of the present work is its correlational nature. We assumed a ‘social causation’ perspective and explored how subjective SES could influence mental health and wellbeing. However, a ‘social selection’ perspective assumes the direction of this relationship is reversed, whereby poor mental health and wellbeing leads to lower SES [70]. There is evidence for both perspectives in the literature, perhaps suggesting a somewhat reciprocal relationship [71]. In other words, the direction examined in the present research seems viable, however the relationships may be bidirectional [see also 72]. Future research using experimental or longitudinal cross-lagged designs is the obvious next step to advance research in this area.

A second limitation concerns our student sample. Given that this research was conducted in the UK, it is not necessarily generalisable to student populations in other countries. Further, as is common within UK Higher Education, our student sample likely included international students, although we did not measure this in the present research. Their international student status may have impacted the extent to which they felt in control, included, and competent. For example, newfound freedoms provided by leaving their home culture and moving far from friends and family may magnify perceived control [73]. International students often experience loneliness and isolation [74], which would likely result in lower perceived inclusion. Finally, international students may be studying in a non-native language, which may lead to reduced perceptions of competence given the additional challenges that come with studying as a non-native speaker [75]. These factors may have a downstream impact on mental health and wellbeing. Future research would benefit from more closely examining the experiences of international students within Higher Education.

### Conclusion

The present research suggests that perceived control, inclusion, and competence may contribute to socioeconomic inequalities in mental health and wellbeing among university students. Lower subjective SES students (*vs*. higher) reported lower perceptions of control, inclusion, and competence, which in turn was associated with poorer mental health and wellbeing. Notably, the present research found that the association between perceived worth and mental health and wellbeing was non-significant in the presence of perceived control, inclusion, and competence. As academic institutions continue to pursue policies to ‘widen participation’, they also have a responsibility to understand how socioeconomic inequalities are perpetuated within university communities. Emerging research in this area marks a first step to improve socioeconomic equality within Higher Education.

## Supporting information

S1 Appendix Demographic characteristics of excluded participant sample. (DOCX)

S2 Appendix Exploratory factor analyses of all scale items. (DOCX)

S3 Appendix Preregistration. (DOCX)

S4 Appendix Data assumptions. (DOCX)

S5 Appendix Sensitivity analyses: Exploring the primary model with additional covariates. (DOCX)

S6 Appendix Further analyses: Exploring a model using objective SES. (DOCX)
