# Supplementary material for: Socioeconomic inequalities in mental health and wellbeing among UK students during the COVID-19 pandemic: Clarifying underlying mechanisms
Source: PLoS One. 2023 Nov 1;18(11):e0292842. doi: 10.1371/journal.pone.0292842 (PMC10619810; doi:10.1371/journal.pone.0292842)
Supplement: S1 Appendix — (DOCX) [file pone.0292842.s001.docx]

**S1 Appendix**

**Table S1. Demographic characteristics for excluded participant sample (*n* = 354)**

| Characteristic | *n* | % |
| --- | --- | --- |
| Gender |  |  |
| Female | 146 | 41.2 |
| Male | 34 | 9.6 |
| Neither female nor male | 13 | 3.7 |
| NA | 161 | 45.5 |
|  |  |  |
| Ethnicity |  |  |
| White | 142 | 40.1 |
| Asian/Asian British | 43 | 12.1 |
| All Other Ethnic Groups | 10 | 2.8 |
| NA | 159 | 44.9 |
|  |  |  |
| Parent/Guardian Household Income |  |  |
| <£15,500 | 6 | 1.7 |
| £15,500 - £24,999 | 6 | 1.7 |
| £25,000 - £40,000 | 27 | 7.6 |
| >£40,000 | 42 | 11.9 |
| NA | 273 | 77.1 |
|  |  |  |
| Recruitment Method |  |  |
| Psychology Participant Pool | 123 | 34.7 |
| Social Media | 189 | 53.4 |
| Prolific | 42 | 11.9 |
