# Supplementary material for: Socioeconomic inequalities in mental health and wellbeing among UK students during the COVID-19 pandemic: Clarifying underlying mechanisms
Source: PLoS One. 2023 Nov 1;18(11):e0292842. doi: 10.1371/journal.pone.0292842 (PMC10619810; doi:10.1371/journal.pone.0292842)
Supplement: S2 Appendix — (DOCX) [file pone.0292842.s002.docx]

S2 Appendix

Exploratory Factor Analysis

**Table S2. Exploratory factor analysis for subjective SES items**

| Items | 1 |
| --- | --- |
| SC1 - Subjective SES Ladder *McArthur Ladder* | 0.82 |
| SC2 - Economic Capital I*ndicate where you would place yourself relative to other students [re.] your income, savings, the value of your family's home and your family's wealth* | 0.82 |
| SC3 - Social Capital  *Indicate where you would place yourself relative to other students [re.] the number of people you know and the status of those people.* | 0.71 |
| SC4 - Cultural Capital  *Indicate where you would place yourself relative to other students [re.] the extent and nature of your cultural interests, activities and hobbies.* | 0.55 |
|  |  |
| Variance Explained | 54% |
| *Note*. EFA was conducted using Principal Axis Factoring with oblimin rotation |  |

**Table S3. Exploratory factor analysis of perceived control, inclusion, perceived worth, and competence items**

| Items | 1 | 2 | 3 | 4 |
| --- | --- | --- | --- | --- |
| **Inclusion** *Since the start of the autumn term, I have felt like other students...* |  |  |  |  |
| Incl3 *…are willing to be friends with me* | **0.87** | -0.04 | -0.01 | 0.00 |
| Incl4 *…are happy for me to belong to their social groups* | **0.86** | -0.01 | 0.00 | -0.01 |
| Incl9 *…accept me* | **0.83** | -0.05 | -0.02 | 0.05 |
| Incl1 *…like me as a person* | **0.82** | 0.03 | 0.01 | -0.02 |
| Incl2 *…feel warmly towards me* | **0.80** | 0.05 | 0.01 | -0.04 |
| Incl7 *…consider me a nice person to have around* | **0.79** | -0.01 | 0.03 | -0.01 |
| Incl6 *…include me in their social activities* | **0.73** | 0.02 | -0.02 | 0.00 |
| Incl5 *…see me as fitting in* | **0.71** | 0.12 | 0.00 | 0.00 |
| Incl8 *…don’t like me (R)* | **0.59** | -0.03 | -0.01 | 0.15 |
|  |  |  |  |  |
| **Perceived Worth** *Since the start of the autumn term, I have felt like other students...* |  |  |  |  |
| PW6 *…look up to me* | -0.07 | **0.88** | -0.02 | 0.01 |
| PW3 *…admire me* | 0.02 | **0.82** | -0.01 | -0.01 |
| PW5 *…consider me a success* | -0.03 | **0.78** | -0.01 | 0.05 |
| PW2 *…think highly of my abilities and talents* | 0.03 | **0.74** | -0.03 | 0.07 |
| PW7 *…consider me a high status individual* | -0.04 | **0.72** | 0.06 | -0.03 |
| PW4 *…see me as an important person* | 0.18 | **0.63** | 0.01 | -0.01 |
| PW1 *…respect my achievements* | 0.18 | **0.59** | 0.08 | -0.03 |
|  |  |  |  |  |
| **Perceived Control**  *Since the start of the autumn term, I have felt like...* |  |  |  |  |
| PC1 *…I have great control over my life* | -0.03 | 0.00 | **0.85** | 0.03 |
| PC3 *…I am able to decide what happens to me* | 0.01 | 0.02 | **0.80** | -0.02 |
| PC4 *…I am able to control the important things in my life* | -0.02 | 0.03 | **0.78** | 0.05 |
| PC6 *...I am free to do what I want* | -0.01 | -0.05 | **0.75** | -0.05 |
| PC2 *…I have great influence on my fate* | 0.00 | 0.04 | **0.73** | -0.01 |
| PC5 *...I am able to control how I spend my time* | 0.08 | -0.08 | **0.64** | 0.03 |
|  |  |  |  |  |
| **Competence** *Since the start of the autumn term, I have felt like...* |  |  |  |  |
| Comp1 *…I have not felt very competent (R)* | -0.01 | 0.00 | -0.02 | **0.82** |
| Comp3 *…I often have not felt very capable (R)* | 0.01 | 0.04 | 0.00 | **0.79** |
| Comp2 *…I have felt a sense of accomplishment from what I do* | 0.04 | 0.08 | 0.15 | **0.51** |
|  |  |  |  |  |
| **Removed Items** |  |  |  |  |
| Comp4 *…I have not had much of a chance to show how capable I am* | 0.04 | -0.14 | 0.11 | 0.42 |
| Comp5 *...people I know have told me I am good at what I do* | 0.05 | 0.25 | 0.09 | 0.23 |
| Comp6 *...I have been able to learn interesting new skills* | 0.04 | 0.02 | 0.19 | 0.28 |
| PW8 *…value my opinions and ideas* | 0.38 | 0.37 | 0.09 | -0.04 |
|  |  |  |  |  |
| **Variance Explained** | 21% | 15% | 13% | 7% |
| *Note*. EFA was conducted using Principal Axis Factoring with oblimin rotation | | | | |

Exploratory Factor Analysis of Mental Health and Wellbeing Items

We conducted exploratory factor analysis (EFA) to determine whether our 12-item mental health and wellbeing scale loaded onto one factor as these scale items have not been used together before. We first used the Kaiser-Meyer-Olkin measure of sampling adequacy (0.92) and Bartlett’s test of sphericity to confirm that factor analysis was appropriate for our data (χ2 (66, 810) = 6053.70, *p* < .001). Initial analysis of Eigenvalues indicated solutions with between two and four factors. These solutions were examined using Principal Factor Analyses (PFA), with both oblimin and promax rotations. The two-factor solution was preferred as there were an insufficient number of primary loadings in the third and fourth factors and further, each additional factor would make our model exponentially more complex as it would act as an additional dependent variable. Two items were eliminated because they did not contribute to a two-factor structure and either (a) did not have a primary factor loading > 0.4 or (b) had a cross-loading > 0.3. The eliminated items were from the PROMIS global physical and mental health scales and provided overall measures of (1) physical health and (2) mental health. A factor analysis of the remaining 10-items indicated that Factor 1 – positive mental health and wellbeing – was comprised of 6 items related to purpose in life, life satisfaction, fulfilment, worthwhileness, happiness, and coping. Loadings ranged from 0.53 to 0.87 and explained 35% of the variance. Factor 2 – negative mental health and wellbeing – was comprised of 4 factors relating to anxiety, stress, worry and emotional exhaustion. Loadings ranged from 0.51 to 0.92 and explained 25% of the variance. See Table S4 for an overview of the items and their associated factor loadings.

**Table S4. Exploratory factor analysis for mental health and wellbeing items**

| Items | 1 | 2 |
| --- | --- | --- |
| **Positive Mental Health and Wellbeing** |  |  |
| WBP3 *Since the start of the autumn term, how often have you felt like you had purpose in your life?* | **0.87** | 0.10 |
| WBP4 *Since the start of the autumn term, how often have you felt like the things you do in your life are worthwhile?* | **0.85** | 0.06 |
| WBP1 *Since the start of the autumn term, how often have you felt satisfied with your life?* | **0.82** | -0.05 |
| WBP5 *Since the start of the autumn term, how often have you felt fulfilled by the activities that you engaged in?* | **0.74** | 0.07 |
| WBP6 *Since the start of the autumn term, how often have you felt like you were effectively coping with important changes that were occurring in your life?* | **0.64** | -0.19 |
| WBP2 *Overall, how happy did you feel yesterday?* | **0.56** | -0.14 |
|  |  |  |
| **Negative Mental Health and Wellbeing** |  |  |
| WBN1 *Since the start of the autumn term, how often have you felt nervous and stressed?* | 0.03 | **0.91** |
| WBN2 *Since the start of the autumn term, how often did you worry?* | 0.06 | **0.85** |
| WBN3 *Since the start of the autumn term, how often have you felt emotionally exhausted?* | -0.17 | **0.69** |
| WBN4 *Overall, how anxious did you feel yesterday?* | -0.17 | **0.50** |
|  |  |  |
| **Removed Items** |  |  |
| *In general, how would you rate your mental health, including your mood and ability to think, since the lockdown began in March 2020?* | 0.57 | -0.36 |
| *In general, how would you rate your physical health since the lockdown began in March 2020?* | 0.39 | -0.08 |
|  |  |  |
| **Variance Explained** | 35% | 22% |
| *Note*. EFA was conducted using Principal Axis Factoring with oblimin rotation. | | |
