# Supplementary material for: Socioeconomic inequalities in mental health and wellbeing among UK students during the COVID-19 pandemic: Clarifying underlying mechanisms
Source: PLoS One. 2023 Nov 1;18(11):e0292842. doi: 10.1371/journal.pone.0292842 (PMC10619810; doi:10.1371/journal.pone.0292842)
Supplement: S3 Appendix — (DOCX) [file pone.0292842.s003.docx]

S3 Appendix

Preregistration

The preregistration details two studies conducted with two samples that addressed separate aims: (1) the impact of policymaker perceptions in the relationship between SES and mental health and wellbeing, and (2) exploring competence as a mediator in the relationship between SES and mental health and wellbeing. Since registering these studies, we decided the two samples should be published separately, with one study for each aim, and the present study focusing on the role of competence as a mediator. This ensures we are not “salami slicing” data (*i.e.,* splitting data from both samples across two papers) [1].

1. Adams NN. Salami Slicing: clarifying common misconceptions for social science early-career researchers. SN Social Sciences. 2022;2(7):88.
