# Supplementary material for: Socioeconomic inequalities in mental health and wellbeing among UK students during the COVID-19 pandemic: Clarifying underlying mechanisms
Source: PLoS One. 2023 Nov 1;18(11):e0292842. doi: 10.1371/journal.pone.0292842 (PMC10619810; doi:10.1371/journal.pone.0292842)
Supplement: S4 Appendix — (DOCX) [file pone.0292842.s004.docx]

S4 Appendix

Data Assumptions

A series of preliminary checks were conducted on the data to determine whether the basic assumptions recommended for structural equation modelling (SEM) were met. We examined the correlations between items on the same latent factor, looking for correlations < 0.5. We assessed extreme collinearity between the items to confirm that none correlated > 0.85 and established that the relationships between the X to M and M to Y variables were linear. Finally, we tested for normality of distribution. The skew and kurtosis was below commonly accepted thresholds for all items (< 3 and < 10, respectively) [1] . However, a Mardia test of multivariate normality of distribution indicated non-normal distribution. For this reason, we employed maximum likelihood (ML) estimation with bootstrapping (5,000 samples) [2].

To assess convergent validity we looked for (1) standardized factor loadings greater than 0.5, (2) composite reliability greater than 0.7, and (3) average variance explained (AVE) greater than 0.5 [3, 4]. Standardised factor loadings, composite reliability and average variance explained (AVE) are presented in Table 4 of the main text. Our data met all three criteria for convergent validity. To assess discriminant validity, we compared the square root of the AVE to the inter-factor correlations. As the square root of the AVE was greater than the inter-factor correlations we were satisfied that discriminant validity was achieved [3]. We also examined the model output to ensure there were no mathematically impossible values that indicate Heywood cases, such as negative variances.

1. Kline RB. Principles and Practice of Structural Equation Modeling. 3rd ed. Little TD, editor. New York: The Guilford Press; 2011.

2. Nevitt J, Hancock GR. Performance of bootstrapping approaches to model test statistics and parameter standard error estimation in structural equation modeling. Structural Equation Modeling. 2001;8(3):353-77.

3. Fornell C, Larcker DF. Evaluating structural equation models with unobservable variables and measurement error. Journal of marketing research. 1981;18(1):39-50.

4. Hair JF, Black WC, Babin BJ, Anderson RE, Tatham R. Multivariate Data Analysis: A Global Perspective. 7th ed. Upper Saddle River: Prentice Hall; 2009.
