# Supplementary material for: Socioeconomic inequalities in mental health and wellbeing among UK students during the COVID-19 pandemic: Clarifying underlying mechanisms
Source: PLoS One. 2023 Nov 1;18(11):e0292842. doi: 10.1371/journal.pone.0292842 (PMC10619810; doi:10.1371/journal.pone.0292842)
Supplement: S5 Appendix — (DOCX) [file pone.0292842.s005.docx]

S5 Appendix

Sensitivity Analyses: Primary Model with Additional Covariates

We included several covariates in the model to determine sensitivity. These covariates were as follows; gender, age, ethnicity, the number of people in their household, and whether they had undertaken a period of self-isolation due to COVID-19 since the beginning of the academic year (yes/no). Model fit was acceptable (χ2 (836, 811) = 2279.97, *p* < .001, RMSEA = 0.046, CFI = 0.926, TLI = 0.918). Indirect effects are reported in Table S5, and path estimates are reported in Fig S1.

**Table S5. Indirect and total effects of hypothesised mediators by model with additional covariates**

| Parameter | Unstandardised Coefficient (b) | SE | Standardised Coefficient (β) |
| --- | --- | --- | --- |
| Indirect Effects |  |  |  |
| SES -> Perceived Control -> Positive Wellbeing | 0.120 | 0.022 | 0.094** |
| SES -> Perceived Control -> Negative Wellbeing | -0.066 | 0.016 | -0.055** |
| SES -> Inclusion -> Positive Wellbeing | 0.071 | 0.017 | 0.056** |
| SES -> Inclusion -> Negative Wellbeing | -0.031 | 0.017 | -0.026 |
| SES -> Perceived Worth -> Positive Wellbeing | 0.006 | 0.017 | 0.005 |
| SES -> Perceived Worth -> Negative Wellbeing | 0.024 | 0.020 | 0.020 |
| SES -> Competence -> Positive Wellbeing | 0.083 | 0.022 | 0.066** |
| SES -> Competence -> Negative Wellbeing | -0.083 | 0.023 | -0.070** |
|  |  |  |  |
| Total Effects |  |  |  |
| SES -> Positive Wellbeing | 0.362 | 0.049 | 0.285** |
| SES -> Negative Wellbeing | -0.219 | 0.045 | -0.183** |
| *Note. SES* indicates subjective SES. *Wellbeing* indicates mental health and wellbeing.  *** p* < .001 | | | |

**Fig S1. Standardised path estimates for primary model with additional covariates**
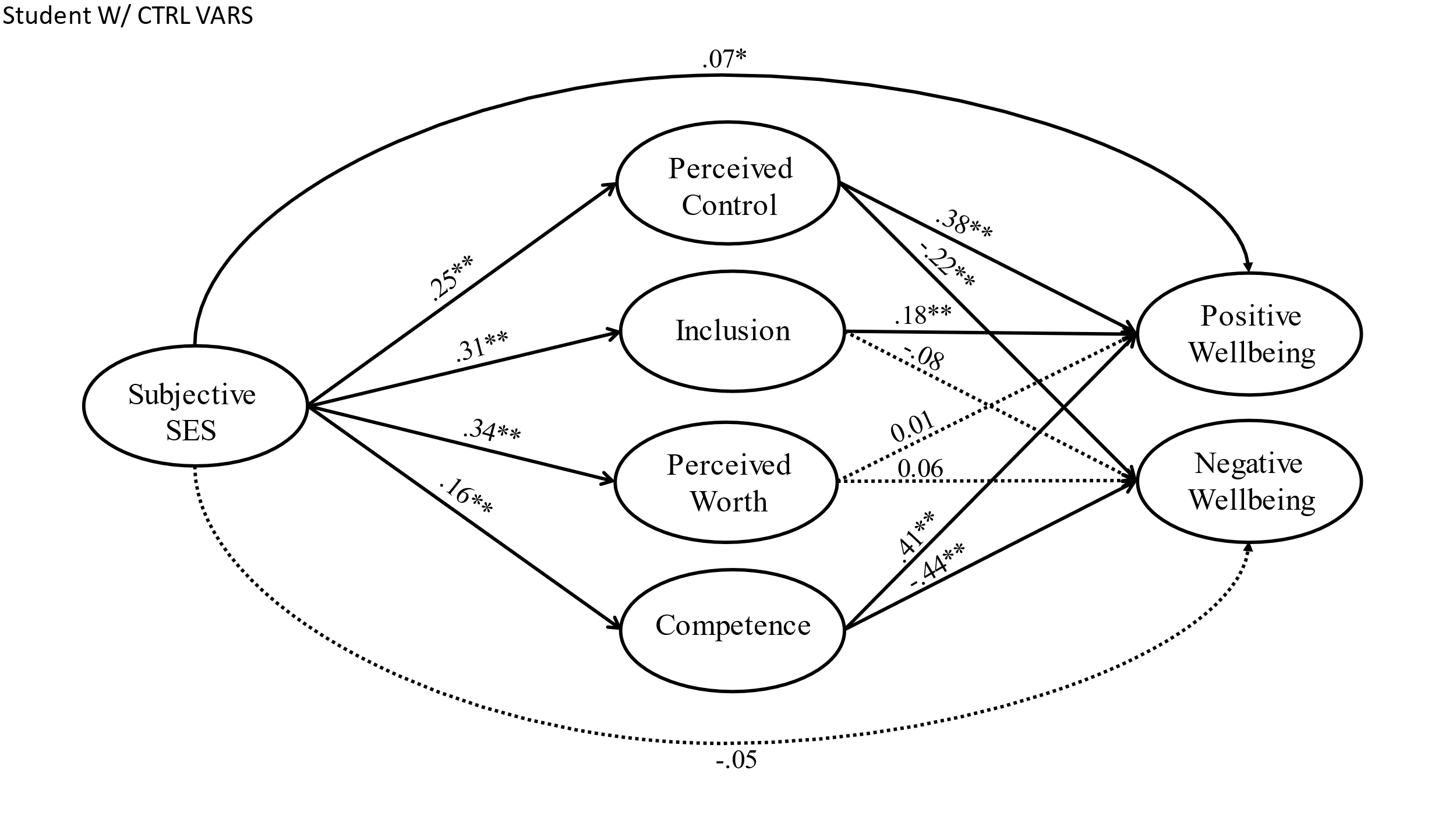


*Note.* Mediators were allowed to covary, as were the two wellbeing variables. *Wellbeing* indicates mental health and wellbeing. Dashed lines are used to emphasise non-significant paths.

***p* < .001; * *p* < .05

**Table S6. Standardised path estimates for additional covariates included in primary model**

|  | Perceived Control | Inclusion | Perceived Worth | Competence | Positive Wellbeing | Negative Wellbeing |
| --- | --- | --- | --- | --- | --- | --- |
| Age | 0.06 | -0.07 | 0.13** | 0.13* | 0.08* | 0.01 |
| Male | 0.13** | 0.01 | 0.08* | 0.19** | -0.08** | -0.16** |
| Asian or Asian British | 0.14** | -0.07* | 0.06 | 0.01 | 0.03 | -0.09* |
| Other Ethnic Groups | -0.02 | -0.01 | 0.09* | -0.01 | 0.03 | -0.02 |
| Experience of isolation | -0.01 | -0.03 | -0.01 | -0.01 | -0.03 | 0.04 |
| No. in HH | 0.01 | 0.12** | 0.00 | -0.04 | 0.06* | -0.05 |
| *Note. Wellbeing* indicates mental health and wellbeing. *Experience of isolation* indicates the respondent undertaken a period of self-isolation due to COVID-19 since the beginning of the academic year. *No. in HH* indicates the number of people living in their household.  *** p* < .01 **p* < .05 | | | | | | |
