# Supplementary material for: Socioeconomic inequalities in mental health and wellbeing among UK students during the COVID-19 pandemic: Clarifying underlying mechanisms
Source: PLoS One. 2023 Nov 1;18(11):e0292842. doi: 10.1371/journal.pone.0292842 (PMC10619810; doi:10.1371/journal.pone.0292842)
Supplement: S6 Appendix — (DOCX) [file pone.0292842.s006.docx]

S6 Appendix

Further Analysis: Model with Objective SES

To determine whether the pattern of results was specific to subjective SES, we conducted further analysis employing objective measures of SES. We used measures of parent/guardian household income, parent/guardian education, and occupation of chief income earner in parent/guardian household. These three items acted as indicator variables for the objective SES latent factor.

Fit was acceptable for this model (χ2 (644, 701) = 1620.26, *p* < .001, RMSEA = 0.047, CFI = 0.939, TLI = 0.934). As per the subjective SES model reported in the main text, in the objective SES model we found significant indirect effects via perceived control, inclusion, and perceived worth. However, we found no significant indirect effect via competence in the relationship between objective SES and mental health and wellbeing. Whilst there was a significant association between competence and both positive and negative mental health and wellbeing, there was no significant association between objective SES and competence. Indirect effects are reported in Table S6, and path estimates are reported in Fig S2.

**Table S7. Indirect and total effects of hypothesised mediators by model with objective SES**

| Parameter | Unstandardised Coefficient (b) | SE | | Standardised Coefficient (β) |
| --- | --- | --- | --- | --- |
| Indirect Effects |  |  | |  |
| Objective SES -> Perceived Control -> Positive Wellbeing | 0.045 | | 0.016 | 0.049* |
| Objective SES -> Perceived Control -> Negative Wellbeing | -0.028 | | 0.012 | -0.032* |
| Objective SES -> Inclusion -> Positive Wellbeing | 0.048 | | 0.013 | 0.053** |
| Objective SES -> Inclusion -> Negative Wellbeing | -0.02 | | 0.012 | -0.023 |
| Objective SES -> Perceived Worth -> Positive Wellbeing | 0.002 | | 0.008 | 0.002 |
| Objective SES -> Perceived Worth -> Negative Wellbeing | 0.011 | | 0.009 | 0.013 |
| Objective SES -> Competence -> Positive Wellbeing | 0.013 | | 0.016 | 0.015 |
| Objective SES -> Competence -> Negative Wellbeing | -0.015 | | 0.018 | -0.018 |
|  |  | |  |  |
| Total Effects |  | |  |  |
| Objective SES -> Positive Wellbeing | 0.161 | | 0.041 | 0.177** |
| Objective SES -> Negative Wellbeing | -0.029 | | 0.038 | -0.034 |
| *Note. Wellbeing* indicates mental health and wellbeing.  *** p* < .001; * *p* < .05 | | | | |

**Fig S2. Standardised path estimates for primary model with objective SES**


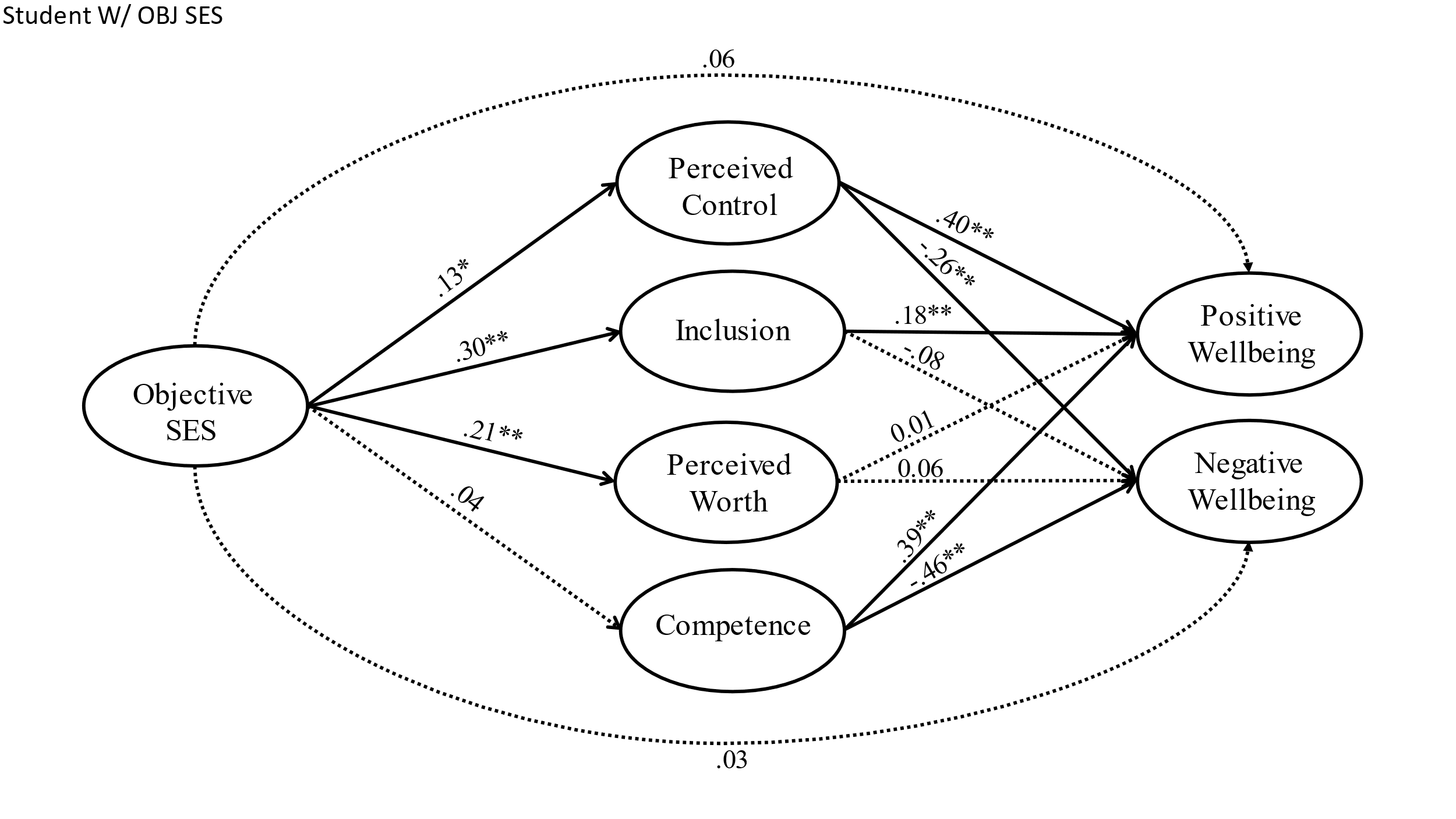


*Note.* Mediators were allowed to covary, as were the two wellbeing variables. *Wellbeing* indicates mental health and wellbeing. Dashed lines are used to emphasise non-significant paths.

***p* < .001; * *p* < .05
